# Clinical Use of Quantitative Analysis of Bone Scintigraphy to Assess the Involvement of Arthritis Diseases in Patients with Joint Symptoms

**DOI:** 10.3390/diagnostics10121000

**Published:** 2020-11-24

**Authors:** Jeong Won Lee, Ki Jin Jung, Sang Mi Lee, Sung Hae Chang

**Affiliations:** 1 Department of Nuclear Medicine, Catholic Kwandong University College of Medicine, International St. Mary’s Hospital, 25, Simgok-ro 100-gil, Seo-gu 22711, Incheon, Korea; sads00@naver.com; 2 Department of Orthopedic Surgery, Soonchunhyang University Cheonan Hospital, 31 Suncheonhyang 6-gil, Dongnam-gu, Cheonan 31151, Chungcheongnam-do, Korea; c89546@schmc.ac.kr; 3 Department of Nuclear Medicine. Soonchunhyang University Cheonan Hospital, 31 Suncheonhyang 6-gil, Dongnam-gu, Cheonan 31151, Chungcheongnam-do, Korea; 4 Division of Rheumatology, Department of Internal Medicine, Soonchunhyang University Cheonan Hospital, 31 Suncheonhyang 6-gil, Dongnam-gu, Cheonan 31151, Chungcheongnam-do, Korea

**Keywords:** arthritis, bone scintigraphy, joint, inflammation, quantitative analysis

## Abstract

We aimed to compare the diagnostic ability of quantitative analysis of bone scintigraphy with that of visual analysis for identifying arthritis disease involvement in patients with joint symptoms. We retrospectively included 93 patients with joint symptoms who underwent Tc-99m methylene diphosphonate bone scintigraphy for evaluating arthritis disease involvement. Bone scintigraphy images were visually and quantitatively analyzed using an in-house software by two reviewers. On quantitative analysis, joint uptake ratio was measured for 64 joints in 14 joints areas. The inter-rater agreement of visual and quantitative analyses was assessed, and diagnostic abilities were compared based on the area under the receiver operating characteristic (ROC) curve (AUC) values. Regarding visual analysis, there was a moderate degree of inter-rater agreement (kappa coefficient of 0.597), while there was a substantial inter-rater agreement (concordance correlation coefficient of 0.987) in the measurement of the joint uptake ratio. The comparisons of ROC curves for the total 5941 joints revealed that the joint uptake ratio had a significantly higher AUC value (0.789) to detect the affected joint than that of the visual analysis (*p* < 0.001). Quantitative analysis using joint uptake ratio showed substantial reproducibility and higher diagnostic ability to detect joints involving arthritis diseases than visual analysis on bone scintigraphy.

## 1. Introduction

Bone scintigraphy using Tc-99m labeled diphosphonate is widely used to evaluate bone and joint diseases given its good availability, low medical cost, and ability to obtain whole-body bone and joint images [1,2,3,4]. Although it has a high sensitivity for the detection of joint involvement in various joint disorders, its potential clinical benefits for evaluating arthritis involvement in individuals with joint symptoms remain contentious [1,5,6,7,8]. This may be due to the existing primary method of bone scintigraphy interpretation, visual assessment, that has involved significant inter-reader discrepancies limiting the general and objective application [6,9]. Therefore, there is a need for an objective quantitative analytic method for bone scintigraphy image interpretations [6,9,10,11]. In patients with bone metastasis, bone scan index, which is a quantitative parameter for the extent of bone metastasis that is automatically calculated using dedicated software, has been introduced as an imaging biomarker of bone metastasis and its use has gradually increased [4,11]. However, when evaluating joint inflammation in patients with arthritic diseases, quantitative analysis has only been performed in a limited joint area, including the sacroiliac, the temporomandibular, and the knee joints [5,6,8], and there have been few attempts at measuring uptake in joints in the whole-body area on a single bone scintigraphy scan [12]. Moreover, the diagnostic abilities of the quantitative parameter of bone scintigraphy and visual analysis to detect joints with arthritic disease involvement in the whole-body area have not been compared.

In this regard, we developed an in-house software that allowed the measurement of a quantitative parameter for joint uptake, joint-to-bone uptake ratio, for each joint in the whole-body area. Using this in-house software, the present study aimed to assess the reproducibility of the joint-to-bone uptake ratio and to compare the diagnostic abilities of the joint-to-bone uptake ratio and visual analysis for detecting joints with arthritic disease involvement in patients with joint symptoms.

## 2. Materials and Methods

### 2.1. Patients

We retrospectively reviewed electronic medical records of patients who underwent bone scintigraphy for an initial work-up of joint pain, tenderness, and/or swelling in our medical center between May 2018 and May 2020. Among them, we enrolled 93 patients based on the following inclusion criteria: patients (1) having bone scintigraphy images suitable for quantitative analysis using our in-house software, specifically, bone scintigraphy images containing whole-body anterior image and spot images of the posterior pelvic area, bilateral hands, and bilateral feet with proper inclusion of all 64 joints in 14 joints areas (bilateral shoulder, sternoclavicular, elbow, knee, sacroiliac, ankle, tarsal, and wrist joints; 10 metatarso-phalangeal (MTP) joints; 10 interphalangeal (IP) joints of foot; 10 metacarpo-phalangeal (MCP) joints; bilateral thumb IP joints; 8 hand proximal IP (PIP) joints; and 8 hand distal IP (DIP) joints), and (2) being aged ≥18 years. Moreover, we excluded patients (1) who had a final clinical diagnosis other than arthritis disease, (2) who were unable to undergo physical examination for arthritic disease involvement in each of the 64 joints, (3) who had recent major traumatic events such as traffic accident or fall-down injury, and (4) who had a history of malignant disease or metabolic bone disease.

Based on the joint symptoms and physical examination, all 64 joints areas were classified as either affected joints (joints with arthritic disease involvement) and nonaffected joints (joints without arthritic disease involvement).

This study was approved by the Institutional Review Board of Soonchunhyang University Cheonan Hospital, (code number: 2018-04-008; 17 April 2018) and the study protocol was in accordance with the ethical standards of the Declaration of Helsinki. The requirement for written informed consent was waived by the Institutional Review Board of Soonchunhyang University Cheonan Hospital due to the retrospective nature of this study.

### 2.2. Bone Scintigraphy

Bone scintigraphy was performed using a dual-head gamma camera (Infinia GP, GE Healthcare, Milwaukee, WI, USA) at 3 h after intravenous injection with Tc-99m methylene diphosphonate at a dose of 740–925 MBq. Continuous acquisition mode was applied at a scanning speed of 12 cm/min using a low-energy general-purpose collimator. Anterior and posterior whole-body images, as well as spot images of the posterior pelvic area, bilateral hands, and bilateral feet, were acquired from all enrolled patients.

### 2.3. Image Analysis

**Figure 1:**
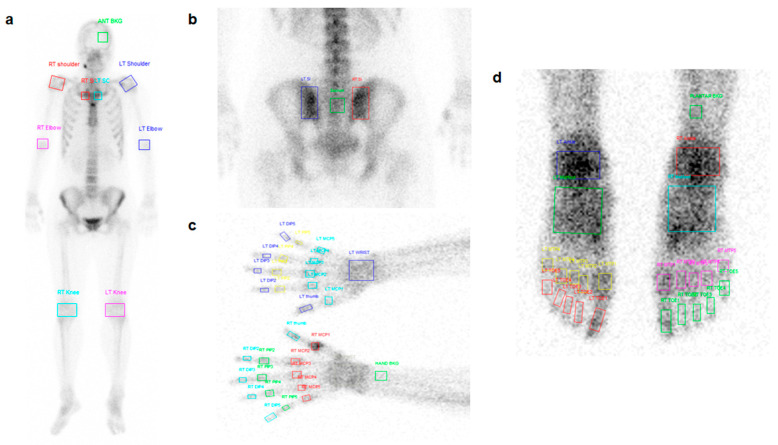
Measurement example of joint uptake ratio in 64 joints of the 14 joint areas (bilateral shoulder, sternoclavicular, elbow, knee, sacroiliac, ankle, tarsal, and wrist joints, 10 metatarso-phalangeal (MTP) joints, 10 interphalangeal (IP) joints of foot, 10 metacarpo-phalangeal (MCP) joints, bilateral thumb IP joints, 8 hand proximal IP (PIP), and 8 hand distal IP (DIP) joints) using an in-house software. We drew a square-shaped region-of-interest for measuring the skull (green), bilateral shoulder (red on right side and blue on left side), sternoclavicular (red on right side and cyan on left side), elbow (pink on right side and blue on left side), and knee joints (cyan on right side and pink on left side) uptake on a whole-body anterior image (**a**), for measuring the sacral bone (green) and bilateral sacroiliac joints (red on right side and blue on left side) uptake on a posterior pelvic spot image (**b**), for measuring the distal radial bone (green), bilateral wrist (blue), MCP (red on right side and cyan on left side), thumb IP (cyan on right side and blue on left side), PIP (green on right side and yellow on left side), and DIP joints (cyan on right side and blue on left side) uptake on a bilateral hands spot image (**c**), and for measuring distal tibial bone (green), bilateral ankle (red on right side and blue on left side), tarsal (cyan on right side and green on left side), MTP (pink on right side and yellow on left side), and toe IP joints (green on right side and red on left side) uptake on a bilateral feet spot image (**d**).

In each patient, we visually and quantitatively assessed the uptake of 64 joints in 14 joint areas on bone scintigraphy images. Among the 5952 joint areas in the 93 patients, 8 knee joint areas and 3 elbow joint areas were excluded for having undergone arthroplasty. Consequently, we included 5941 joints in image analysis. Regarding visual analysis, two nuclear medicine physicians who were blinded to the patients’ clinical information independently classified each joint using a three-point grading system as follows: grade 1, joints with mildly increased radiotracer uptake; grade 2, joints with moderately increased radiotracer uptake; and grade 3, joints with intensely increased radiotracer uptake. Regarding quantitative analysis, two nuclear medicine physicians independently measured the joint-to-bone uptake ratio (joint uptake ratio) for each joint. Using an in-house software, a square-shaped region-of-interest was drawn for each joint and the reference bone uptake. Afterwards, the mean values of the joints and the reference bone uptake and the joint-to-bone uptake ratio were automatically calculated (Figure 1). Using a whole-body anterior image, we measured the skull uptake as a reference bone uptake, as well as the uptake of the bilateral shoulder, sternoclavicular, elbow, and knee joints. On a posterior pelvic spot image, we measured the sacral bone uptake as a reference bone uptake, as well as the uptake of the bilateral sacroiliac joints. On a bilateral feet spot image, we measured the distal tibial bone uptake as a reference bone uptake, as well as uptake of the bilateral ankle, tarsal, MTP, and toe IP joints. On a bilateral hands spot image, we measured the distal radial bone uptake as a reference bone uptake, as well as uptake of the bilateral wrist, MCP, thumb IP, PIP, and DIP joints.

Of the 5941 joint areas included in the bone scintigraphy image analysis, joint X-ray imaging was also performed in 2356 joint areas (39.6%) of 68 patients. Two physicians visually assessed all joint X-ray images and determined the joints with bone erosion among those 2356 joints. 

### 2.4. Statistical Analysis

To evaluate the inter-rater agreement in the grading joints between the two readers, the weighted Cohen’s Kappa coefficient was derived. To assess the inter-reader reproducibility of the measured joint uptake ratio between the two readers, concordance correlation coefficient was calculated. Student’s t-test and Mann–Whitney test were performed to compare differences in the joint uptake ratios between the affected and nonaffected joints and between joints with bone erosions and without bone erosions on X-ray images. The diagnostic abilities of visual analysis of both readers and the joint uptake ratio were evaluated based on the area under the receiver operating characteristic (ROC) curve (AUC) values. On comparisons of AUC values between visual analysis and joint uptake ratio, the Bonferroni adjustment was applied for multiple comparisons. Using the optimal cut-off values determined by ROC curve analysis, the sensitivity, specificity, positive predictive value, negative predictive value, and accuracy of the joint uptake ratio for detecting affected joint were assessed. All statistical analyses were performed using MedCalc Statistical Software version 19.3.1 (MedCalc Software Ltd., Ostend, Belgium), and statistical significance was set at a *p*-value of <0.05.

## 3. Results

### 3.1. Patient Characteristics

Among the 93 enrolled patients, there were 66 women (71.0%) and 27 men (29.0%). The median age of the patients was 56 years (range, 18–81 years). The clinical diagnoses of the patients were osteoarthritis in 48 patients (51.6%), rheumatoid arthritis in 41 patients (44.1%), ankylosing spondylitis in two patients (2.2%), psoriatic arthritis in one patient (1.1%), and sarcoidosis in one patient (1.1%). The median number of affected joints was five (range, 1–31 joints). Table 1 summarizes the proportions of affected joints for each joint area. Among the total 5941 joints, 728 joints (12.3%) were classified as affected joints, which were mainly located in the wrist and hand area.

### 3.2. Inter-Rater Agreement

**Figure 2:**
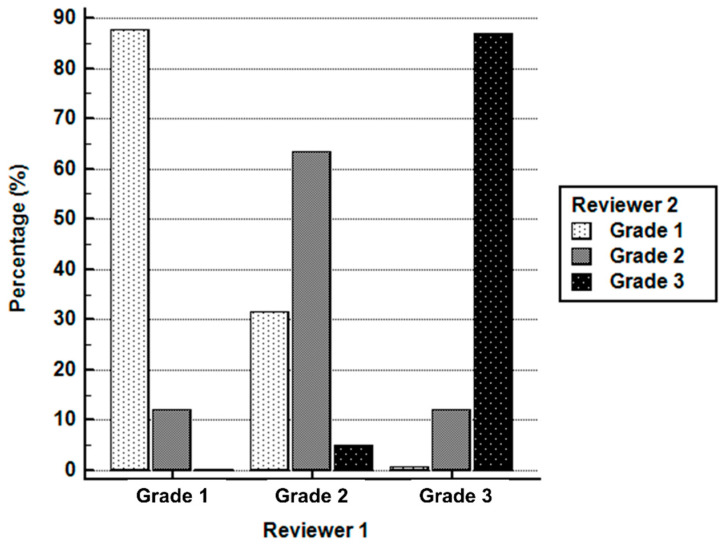
Inter-rater agreement in the grading joints on bone scintigraphy between two readers.

Regarding visual analysis, there was a moderate inter-rater agreement in the grading joints between both readers. The exact inter-reader agreement of the joint grade was 81.5% (4844 out of 5941 joints). Analysis using the weighted Cohen’s kappa yielded a kappa coefficient of 0.597 (95% confidence interval (CI), 0.575–0.619). Among three grades, only 63.4% agreement was found for grade 2 between both readers, while grade 1 and grade 3 showed the agreement of 87.8% and 87.1%, respectively (Figure 2).

Regarding quantitative analysis, the measured joint uptake ratio showed a substantial inter-rater agreement between both readers. The inter-reader concordance correlation coefficient to measure the joint uptake ratio was 0.987 (95% CI, 0.983–0.991).

### 3.3. Comparison of the Joint Uptake Ratio

**Figure 3:**
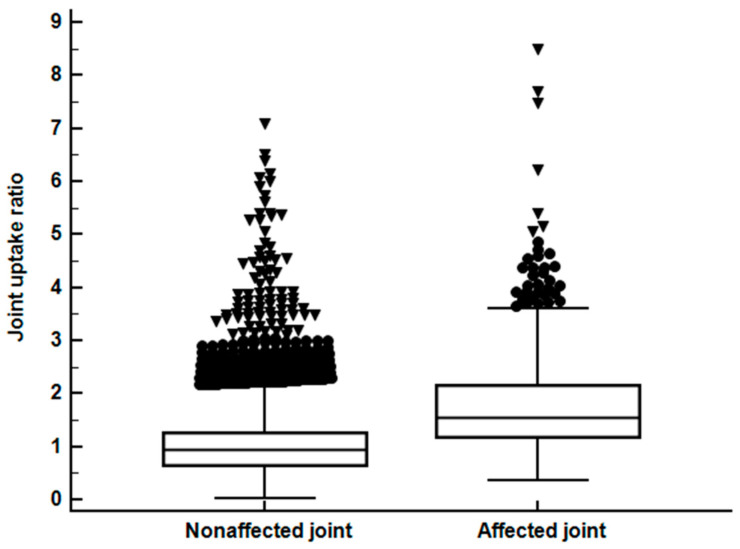
Distribution of the joint uptake ratio of 5213 nonaffected joints and 728 affected joints.

The joint uptake ratios between the affected and nonaffected joints were compared for each joint area (Table 1). Joint uptake ratio of all affected joints (1.79 ± 0.95) was significantly higher than that of all nonaffected joints (1.06 ± 0.64; *p* < 0.001; Figure 3). The joint uptake ratios of affected joints in the elbow, knee, ankle, tarsal, MTP, toe IP, wrist, thumb IP, MCP, hand PIP, and hand DIP joint areas were significantly higher than those of the nonaffected joints (*p* < 0.05; Table 1; Supplementary Figure S1). Contrastingly, there was no significant differences in the joint uptake ratio between the affected and nonaffected joints in the shoulder, sternoclavicular, and sacroiliac joint areas (*p* > 0.05; Table 1; see Supplementary Figure S1 online).

Of the 2356 joint areas assessed by X-ray images, 162 joints (7.4%) were defined as joints with bone erosions. Joint uptake ratio of these joints with bone erosions (1.60 ± 1.00) were significantly higher than joints without bone erosions (1.11 ± 0.59; *p* < 0.001)

### 3.4. Comparison of Diagnostic Ability

**Figure 4:**
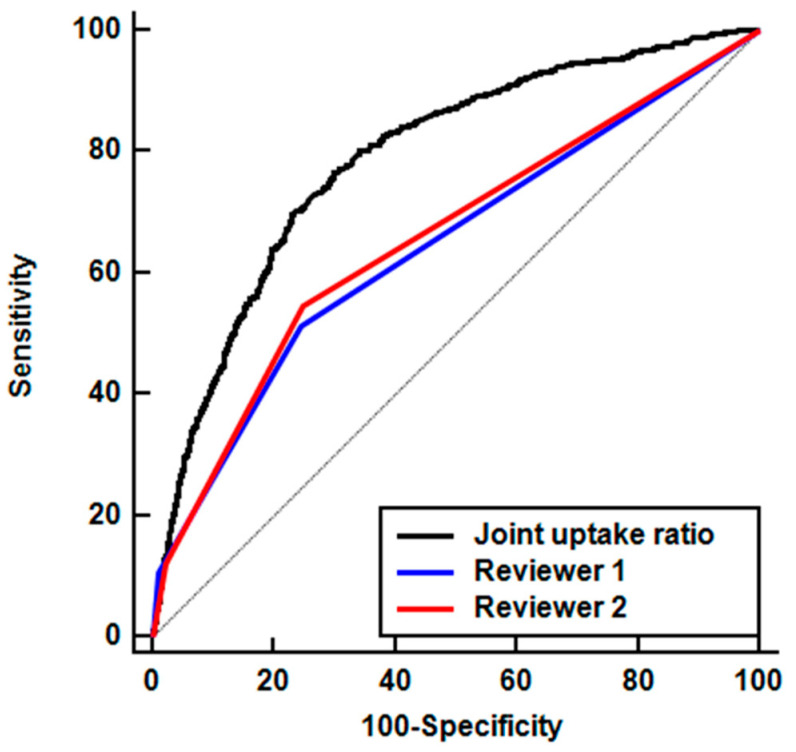
Comparison of receiver operating characteristic curves for the visual analysis of two readers (Reviewer 1 and Reviewer 2) and the joint uptake ratio in detecting affected joints among the 5941 joints.

The AUC values between the joint uptake ratio and visual analysis results of both readers were compared after the Bonferroni correction (Table 2). For all included joints, the joint uptake ratio had a significantly higher AUC value (0.789; 95% CI, 0.778–0.799) for detecting the affected joint than that of visual analysis (*p* < 0.001 for both; Figure 4). Regarding the 14 joint areas, the joint uptake ratio revealed significantly higher AUC values than visual analysis of both readers in the knee, ankle, MTP, toe IP, wrist, MCP, and hand PIP joints (*p* < 0.017 for all; Table 2; Supplementary Figure S2). On the other hand, there were no significant between-analysis differences in the AUC values for the shoulder, sternoclavicular, elbow, sacroiliac, thumb, IP, and hand DIP joints (*p* > 0.017; Table 2; see Supplementary Figure S2 online).

The diagnostic ability of joint uptake ratio for detecting affected joints was assessed using the optimal cut-off values identified by ROC curve analysis (Table 3). Using a cut-off values of 1.20, the joint uptake ratio showed a sensitivity of 75.1% (95% CI, 71.8–78.2%), specificity of 70.7% (95% CI, 69.5–71.9%), positive predictive value of 26.3% (95% CI, 25.2–27.3%), negative predictive values of 95.3% (95% CI, 94.7–95.9%), and accuracy of 71.2% for detecting the affected joints among all 5941 joints. Among 14 joint areas, the joint uptake ratio showed a high sensitivity of ≥80.0% for detecting affected joints in the shoulder, sternoclavicular, knee, sacroiliac, tarsal, and MCP joint areas, and it showed a high specificity of >80.0% in the elbow, ankle, wrist, hand PIP, and hand DIP joints. Furthermore, for all joint areas other than the wrist and hand PIP joints, the joint uptake ratio showed a high negative predictive value of >90.0% for detecting affected joints.

## 4. Discussion

In the present study, we developed an in-house software for quantitative assessment of the whole-body joint uptake on bone scintigraphy images. Currently, visual analysis has been the primary analytic method for bone scintigraphy. However, visual analysis is subjective and is dependent on the clinical experience of the reader, and previous studies have reported a moderate degree of inter-rater agreement in the interpretation of bone scintigraphy images with kappa values between 0.48–0.54 [9,13,14]. Similarly, we observed a moderate degree of agreement (kappa value of 0.597) between both the readers in joint uptake classification. In contrast, the joint uptake ratio measured in our software showed substantial between-reader measurement agreement with a concordance correlation coefficient of 0.987, suggesting the use of the joint uptake ratio as a reproducible objective parameter for bone scintigraphy.

There have been few studies comparing the diagnostic ability between visual and quantitative analysis for assessing active arthritis in only the sacroiliac and temporomandibular joints, which failed to show a superiority of quantitative parameters over visual analysis [6,8]. In the present study, we tried to compare the diagnostic ability of joint uptake ratio with visual analysis not only for total whole-body joints but also for each 14 joint areas. Regarding the total joints, affected joints showed significantly higher joint uptake ratios than nonaffected joints. Furthermore, joints with bone erosions on X-ray images showed significantly higher joint uptake ratios than other joints, suggesting the correlation between joint uptake ratio and joint damage. More importantly, compared with visual assessment, joint uptake ratio showed a significantly higher diagnostic ability for detecting joints with arthritic disease involvement. On the other hand, regarding the evaluation of 14 joint areas, the results differed across the joint areas. Regarding shoulder and sternoclavicular joints, both the joint uptake ratio and visual analysis showed a low diagnostic ability of AUC less than 0.700 without a significant difference of AUC between them. Previous studies have reported increased uptake in the shoulder and sternoclavicular joint areas on bone scintigraphy without clinically active arthritis [15,16]. Similarly, this study observed no significant differences in the joint uptake ratio between affected and nonaffected joints in these joint areas. Therefore, bone scintigraphy could have limited clinical value for evaluating active arthritis in the shoulder and sternoclavicular joints. For the elbow, sacroiliac, thumb IP, and hand DIP joint areas, there were no significant differences in the diagnostic ability between visual assessment and joint uptake ratio, whereas joint uptake ratio showed a high diagnostic ability for the knee, ankle, tarsal, wrist, MCP, and hand PIP joint areas. Our findings implied that quantitative parameters measured on bone scintigraphy might have better clinical utility in the assessment of arthritis disease compared with visual analysis, but the quantitative analytic method should be applied based on the sites of joint symptoms. However, given the limited value of bone scintigraphy in the differential diagnosis of arthritic diseases and that it can present increased joint uptake even in chronic arthritis [1,12,17,18], there is a need for further studies on patients with a single disease to validate the clinical utility of our quantitative analytic method. Particularly, with the establishment of cut-off joint uptake ratio for defining affected joints, our quantitative analytic method could be useful to exclude patients without active arthritis among those with persistent multiple joint pains, or to objectively assess active arthritis among the whole-body joints upon initial work-up of rheumatoid arthritis.

Although the joint uptake ratio showed significantly higher diagnostic ability than visual analysis, it had moderate sensitivity and specificity for detecting arthritic disease involvement in total joints, and quite a number of nonaffected joints, even in joint areas other than the shoulder and sternoclavicular joint areas, have shown increased uptake on bone scintigraphy similar to uptake of affected joints. This finding could be attributed to several factors. Subclinical joint damage, which is also known to show abnormal findings on other imaging examinations such as magnetic resonance imaging (MRI), could contribute to increased uptake on nonaffected joints [19,20]. In addition, arthropathy in the knee, ankle, and foot joint areas could affect the condition of the overlying and contralateral joint areas due to the changes in mechanical load, which could lead to increase the joint uptake [21,22]. Although uptake in nonaffected joints on bone scintigraphy could be merely considered as a false positive finding, it might be worth investigating the clinical significance of nonaffected joints uptake in future studies.

Given the difficulty in the precise and objective clinical assessment of whole-body joints through physical examination, several imaging modalities, including plain radiography, ultrasonography, MRI, positron emission tomography/computed tomography (PET/CT), and bone scintigraphy, have been used to evaluate arthritic disease involvement [17,23,24,25]. Plain radiography is the most widely used imaging examination; however, it has low sensitivity for small and subtle pathological findings [23,26]. Ultrasonography and MRI have additional benefits in the clinical assessment of joint lesions; however, evaluating whole-body joints is not easily applicable for both imaging modalities and their interpretation is still mainly dependent on visual analysis [23,24,26,27]. Recently, the clinical use of PET/CT using F-18 sodium fluoride and F-18 fluorodeoxyglucose has been studied in patients with arthritic diseases [25,28]. PET/CT imaging has higher sensitivity and resolution than bone scintigraphy and allows quantitative analysis using standardized uptake value, but the clinical implication of PET/CT imaging still needs further validation in patients with joint symptoms [28,29,30]. Although bone scintigraphy has good availability and low medical cost and can evaluate whole-body joints with high sensitivity, its clinical benefit in the evaluation of joint inflammation using conventional visual analysis remains unclear [17,31]. However, the results of our study demonstrated that with the use of our in-house software for quantitative analysis, diagnostic ability of bone scintigraphy for determining affected joints could be improved, which may bring additional clinical benefit and enhance the clinical use of bone scintigraphy. Nevertheless, before considering the general application of our method in patients with arthritis diseases, there is an inherent drawback of our quantitative analytic method to overcome. In patients with long-lasting active arthritis, joints could be severely distorted and show anatomical deformity, in which a reliable quantitative analysis with anterior and posterior bone scintigraphy images may be difficult. The use of restraints such as limb positioning device during imaging might help to acquire optimal images for these patients [32]. Furthermore, with the advancement of medical technology, whole-body single-photon emission computed tomography/CT with ultra-fast protocol has started to be used in recent studies [33,34], and it could be a promising imaging tool for quantifying joint uptake in those patients. 

This study has several limitations. First, this was a retrospective single-center study with a relatively small number of patients with various types of arthritic diseases. Therefore, our findings should be further validated in patients with a single disease entity. Second, the enrolled patients had a broad age range and an uneven sex distribution. Previous studies have reported that age and sex can affect the uptake of sacroiliac and temporomandibular joints [35,36]; thus, joint uptake ratio in other joint areas might be also influenced by age and sex, which could further affect the diagnostic ability of bone scintigraphy. Third, in diseases with extra-articular manifestations, such as psoriatic arthritis and sarcoidosis, the degree of extra-articular disease burden might affect the interpretation of bone scintigraphy. Finally, given the retrospective study design, we could not compare the bone scintigraphy results with findings of other imaging modalities such as joint ultrasonography and MRI and could not determine whether the quantitative analytic method in bone scintigraphy has a superior clinical value in management planning compared with visual analysis.

## 5. Conclusions

In conclusion, we were able to measure quantitative parameters on bone scintigraphy, the joint uptake ratio, for whole-body 14 joint areas in patients with joint symptoms. Joint uptake ratio showed substantial agreement on measurements between two readers, and these were significantly higher in affected joints than in nonaffected joints. Furthermore, the joint uptake ratio showed significantly higher AUC values compared with visual analysis in the knee, ankle, MTP, toe IP, wrist, MCP, and finger PIP joints, as well as the total whole-body joints. This quantitative analytic method might contribute to the assessment of arthritic disease involvement in joints; however, there is a need for further studies to validate its clinical value.

## Figures and Tables

**Table 1:** The proportions and joint uptake ratios of affected and nonaffected joints in the 93 enrolled patients.

Joints	Total Joint		Affected Joint		Nonaffected Joint		*p*-Value ^†^
	No.	Joint Uptake Ratio *	No. (%)	Joint Uptake Ratio *	No. (%)	Joint Uptake Ratio *	
Shoulder	186	1.57 ± 0.73	30 (16.1%)	1.73 ± 0.76	156 (83.9%)	1.54 ± 0.72	0.094
Sternoclavicular	186	2.95 ± 1.40	5 (2.7%)	3.17 ± 0.21	181 (97.3%)	2.95 ± 1.42	0.291
Elbow	183	0.99 ± 0.50	20 (10.9%)	1.48 ± 0.95	163 (89.1%)	0.93 ± 0.38	0.036
Knee	178	1.39 ± 0.60	37 (20.8%)	2.02 ± 0.80	141 (79.2%)	1.23 ± 0.41	<0.001
Sacroiliac	186	1.10 ± 0.18	4 (2.2%)	1.23 ± 0.09	182 (97.8%)	1.10 ± 0.18	0.056
Ankle	186	1.70 ± 0.62	27 (14.5%)	2.49 ± 0.75	159 (85.5%)	1.57 ± 0.48	<0.001
Tarsal	186	1.51 ± 0.53	15 (8.1%)	2.36 ± 0.80	171 (91.9%)	1.43 ± 0.42	<0.001
MTP	930	0.92 ± 0.48	64 (6.9%)	1.38 ± 1.06	866 (93.1%)	0.88 ± 0.39	<0.001
Toe IP	930	0.63 ± 0.26	10 (1.1%)	0.78 ± 0.18	920 (98.9%)	0.63 ± 0.26	0.017
Wrist	186	2.32 ± 0.98	100 (53.8%)	2.76 ± 1.07	86 (46.2%)	1.82 ± 0.50	<0.001
Thumb IP	186	1.21 ± 0.44	26 (14.0%)	1.44 ± 0.52	160 (86.0%)	1.17 ± 0.41	0.007
MCP	930	1.30 ± 0.52	179 (19.2%)	1.73 ± 0.69	751 (80.8%)	1.20 ± 0.41	<0.001
PIP	744	1.03 ± 0.46	158 (21.2%)	1.37 ± 0.67	586 (78.8%)	0.94 ± 0.33	<0.001
DIP	744	0.90 ± 0.45	53 (7.1%)	1.63 ± 0.97	691 (92.9%)	0.84 ± 0.32	<0.001
Total	5941	1.15 ± 0.72	728 (12.3%)	1.79 ± 0.95	5213 (87.7%)	1.06 ± 0.64	<0.001

* Mean ± standard deviation; † *p*-values for comparisons of uptake ratios between affected and nonaffected joints; DIP, distal interphalangeal joints; IP, interphalangeal joints; MCP, metacarpo-phalangeal joints; MTP, metatarso-phalangeal joints; PIP, proximal interphalangeal joints.

**Table 2:** Comparisons of the area under the receiver operating characteristic curve values between the joint uptake ratio and the visual analysis results of two readers.

Joint	The Area under the Receiver Operating Characteristic Curve				
	Joint Uptake Ratio (95% CI)	Reviewer 1 (95% CI)	*p*-Value ^†^	Reviewer 2 (95% CI)	*p*-Value ^‡^
Shoulder	0.597 (0.523–0.668)	0.661 (0.588–0.729)	0.332	0.564 (0.490–0.637)	0.621
Sternoclavicular	0.639 (0.565–0.708)	0.509 (0.435–0.583)	0.364	0.655 (0.582–0.723)	0.861
Elbow	0.644 (0.570–0.713)	0.536 (0.461–0.610)	0.191	0.502 (0.427–0.576)	0.228
Knee	0.828 (0.765–0.881)	0.664 (0.589–0.733)	0.001	0.657 (0.582–0.726)	<0.001
Sacroiliac	0.780 (0.713–0.837)	0.713 (0.642–0.777)	0.422	0.512 (0.438–0.586)	0.143
Ankle	0.848 (0.788–0.896)	0.648 (0.575–0.717)	<0.001	0.570 (0.496–0.642)	<0.001
Tarsal	0.877 (0.821–0.921)	0.746 (0.677–0.807)	0.028	0.787 (0.721–0.843)	0.016
MTP	0.751 (0.722–0.778)	0.610 (0.577–0.641)	<0.001	0.545 (0.512–0.577)	<0.001
Toe IP	0.720 (0.689–0.748)	0.555 (0.522–0.587)	0.009	0.569 (0.537–0.602)	0.015
Wrist	0.850 (0.791–0.898)	0.599 (0.525–0.670)	<0.001	0.607 (0.533–0.678)	<0.001
Thumb IP	0.665 (0.592–0.732)	0.528 (0.454–0.602)	0.115	0.547 (0.472–0.620)	0.101
MCP	0.791 (0.763–0.816)	0.601 (0.569–0.633)	<0.001	0.626 (0.594–0.657)	<0.001
PIP	0.764 (0.732–0.794)	0.608 (0.572–0.643)	<0.001	0.609 (0.573–0.644)	<0.001
DIP	0.764 (0.732–0.794)	0.784 (0.753–0.813)	0.538	0.788 (0.757–0.817)	0.366
Total	0.789 (0.778–0.799)	0.644 (0.632–0.656)	<0.001	0.658 (0.646–0.670)	<0.001

† *p*-value for comparison between the joint uptake ratio and the results of reviewer 1. Statistically significant for *p*-value < 0.017; ‡ *p*-value for comparison between the joint uptake ratio and the results of reviewer 2. Statistically significant for *p*-value <0.017; CI, confidence interval; DIP, distal interphalangeal joints; IP, interphalangeal joints; MCP, metacarpo-phalangeal joints; MTP, metatarso-phalangeal joints; PIP, proximal interphalangeal joints.

**Table 3:** The diagnostic ability of joint uptake ratio for detecting affected joints.

Joint	Cut-Off Joint Uptake Ratio	Sensitivity (%) (95% CI)	Specificity (%) (95% CI)	PPV (%) (95% CI)	NPV (%) (95% CI)	Accuracy (%)
Shoulder	1.36	80.0 (61.4–92.3)	56.4 (48.2–64.3)	26.1 (21.5–31.2)	93.6 (87.6–96.8)	60.2
Sternoclavicular	3.00	100.0 (47.8–100.0)	59.7 (52.1–66.9)	6.4 (5.4–7.6)	100.0 (94.9–100.0)	57.5
Elbow	1.41	50.0 (27.2–72.8)	92.6 (87.5–96.1)	45.5 (29.3–62.6)	93.8 (90.7–92.9)	88.0
Knee	1.44	81.1 (64.8–92.0)	70.2 (61.9–77.6)	41.7 (34.7–49.0)	93.4 (87.8–96.5)	72.5
Sacroiliac	1.13	100.0 (39.8–100.0)	61.5 (54.1–68.6)	5.4 (4.5–6.4)	100.0 (95.3–100.0)	62.4
Ankle	1.97	74.1 (53.7–88.9)	86.2 (79.8–91.1)	47.6 (36.8–58.7)	95.1 (91.2–97.4)	84.4
Tarsal	1.69	93.3 (68.1–99.8)	73.1 (65.8–79.6)	23.3 (18.7–28.7)	99.2 (94.9–99.9)	74.7
MTP	1.05	65.6 (52.7–77.1)	73.9 (70.8–76.8)	15.7 (13.1–18.6)	96.7 (95.4–97.7)	73.3
Toe IP	0.78	70.0 (34.8–93.3)	76.0 (73.1–78.7)	3.1 (2.0–4.6)	99.6 (98.9–99.8)	75.9
Wrist	2.20	77.0 (67.5–84.8)	84.9 (75.5–91.7)	85.1 (77.3–90.5)	73.7 (66.6–79.8)	80.6
Thumb IP	1.28	65.3 (44.3–82.8)	67.5 (59.7–74.7)	24.6 (18.6–31.9)	92.3 (87.5–95.4)	67.2
MCP	1.28	86.0 (80.1–90.8)	66.2 (61.3–69.3)	37.7 (34.2–39.5)	95.2 (93.1–96.6)	70.0
PIP	1.17	62.0 (54.0–69.6)	80.7 (77.3–83.8)	46.2 (41.1–51.4)	88.6 (86.4–90.5)	76.7
DIP	1.45	52.8 (38.6–66.7)	96.4 (94.7–97.6)	52.8 (41.4–64.0)	96.4 (95.2–97.3)	93.3
Total	1.20	75.1 (71.8–78.2)	70.7 (69.5–71.9)	26.3 (25.2–27.3)	95.3 (94.7–95.9)	71.2

CI, confidence interval; DIP, distal interphalangeal joints; IP, interphalangeal joints; MCP, metacarpo-phalangeal joints; MTP, metatarso-phalangeal joints; NPV, negative predictive value; PIP, proximal interphalangeal joints; PPV, positive predictive value.

## Supplementary Materials

The following are available online at https://www.mdpi.com/2075-4418/10/12/1000/s1. Figure S1: Distribution of the joint uptake ratio of nonaffected joints and affected joints in shoulder (A), sternoclavicular (B), elbow (C), knee (D), sacroiliac (E), ankle (F), tarsal (G), metatarso-phalangeal (MTP) (H), toe interphalangeal (IP) (I), wrist (J), thumb IP (K), metacarpo-phalangeal (MCP), (L), finger proximal interphalangeal (PIP) (M), and finger distal interphalangeal (DIP) (N) joints, Figure S2: Comparison of receiver operating characteristic curves for the visual analysis of two readers (Reviewer 1 and Reviewer 2) and the joint uptake ratio in detecting affected joints on shoulder (A), sternoclavicular (B), elbow (C), knee (D), sacroiliac (E), ankle (F), tarsal (G), metatarso-phalangeal (MTP) (H), toe interphalangeal (IP) (I), wrist (J), thumb IP (K), metacarpo-phalangeal (MCP), (L), finger proximal interphalangeal (PIP) (M), and finger distal interphalangeal (DIP) (N) joints.
