# Supplementary material for: Clinical Use of Quantitative Analysis of Bone Scintigraphy to Assess the Involvement of Arthritis Diseases in Patients with Joint Symptoms
Source: Diagnostics (Basel). 2020 Nov 24;10(12):1000. doi: 10.3390/diagnostics10121000 (PMC7761348; doi:10.3390/diagnostics10121000)

Supplementary Figure. S1. Distribution of the joint uptake ratio of nonaffected joints and affected joints in shoulder (**A**), sternoclavicular (**B**), elbow (**C**), knee (**D**), sacroiliac (**E**), ankle (**F**), tarsal (**G**), metatarso-phalangeal (MTP) (**H**), toe interphalangeal (IP) (**I**), wrist (**J**), thumb IP (**K**), metacarpo-phalangeal (MCP), (**L**), finger proximal interphalangeal (PIP) (**M**), and finger distal interphalangeal (DIP) (**N**) joints.

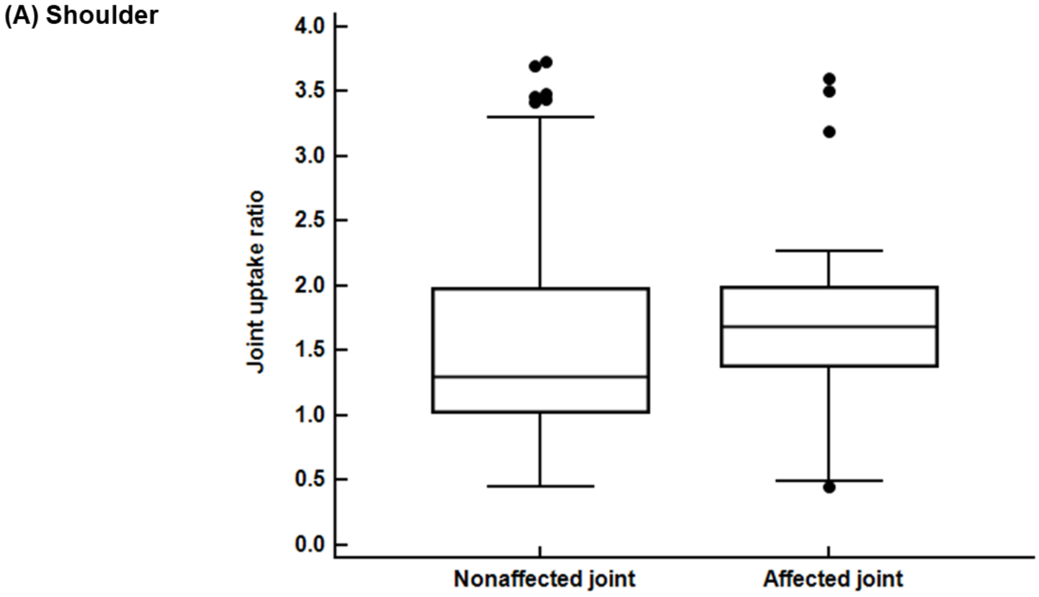

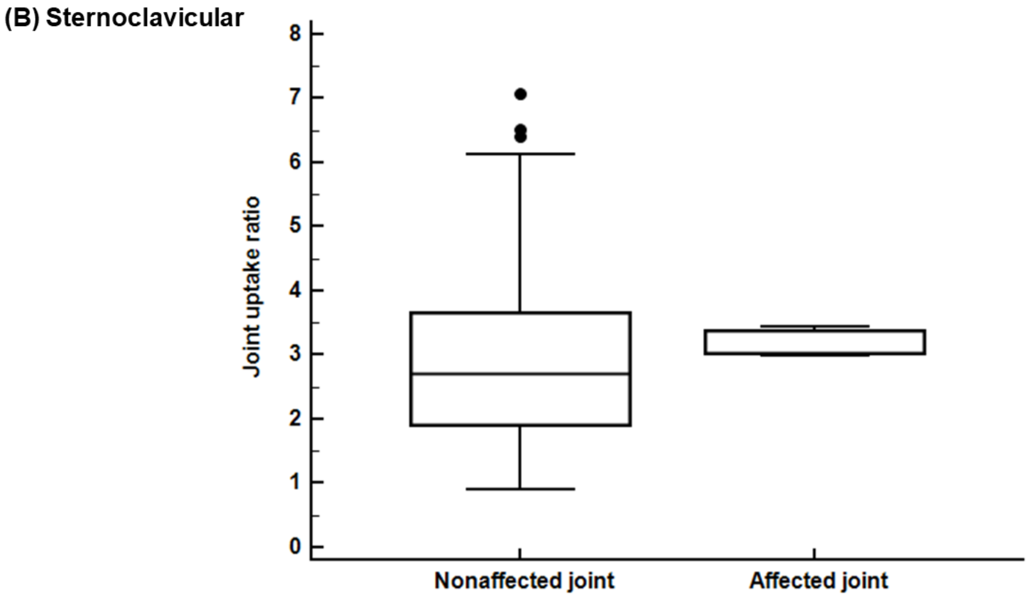

(C) Elbow

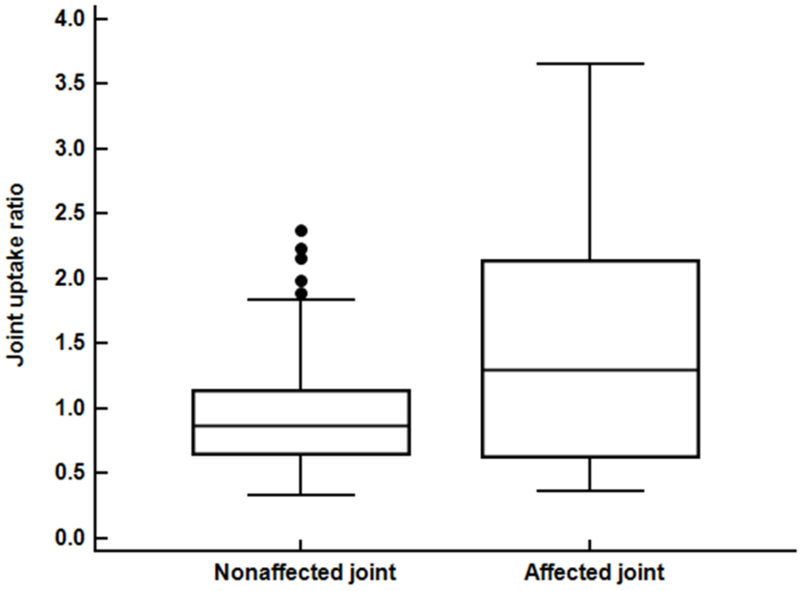

(D) Knee

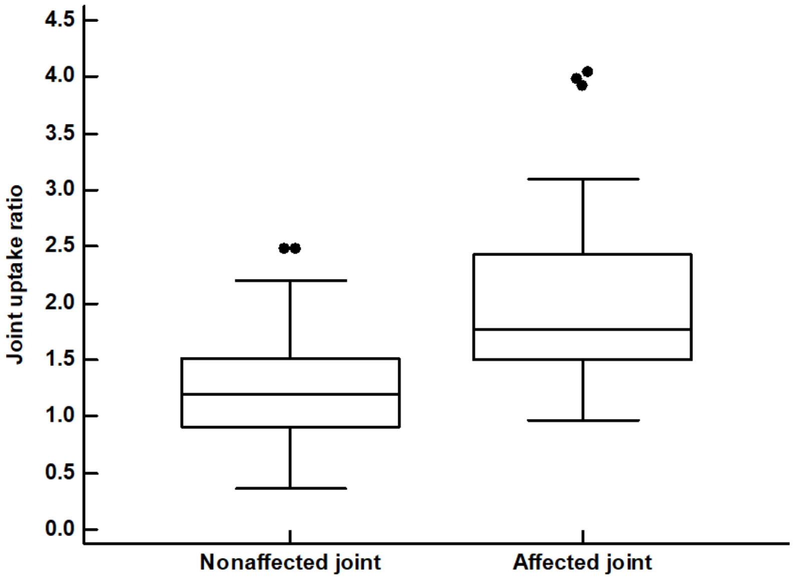

(E) Sacroiliac

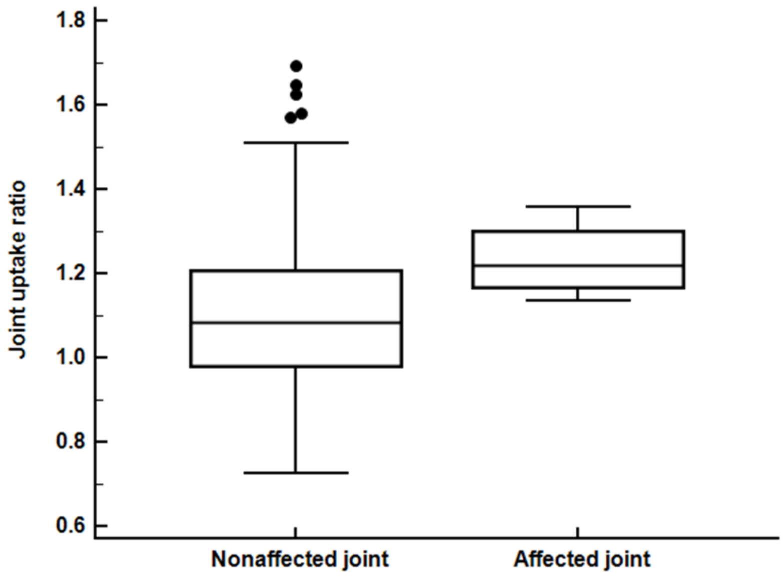

(F) Ankle

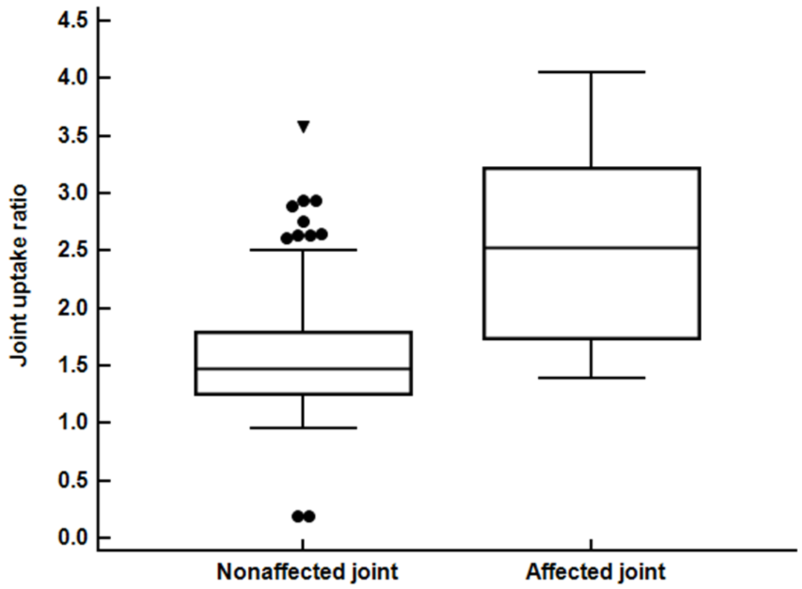

(G) Tarsal

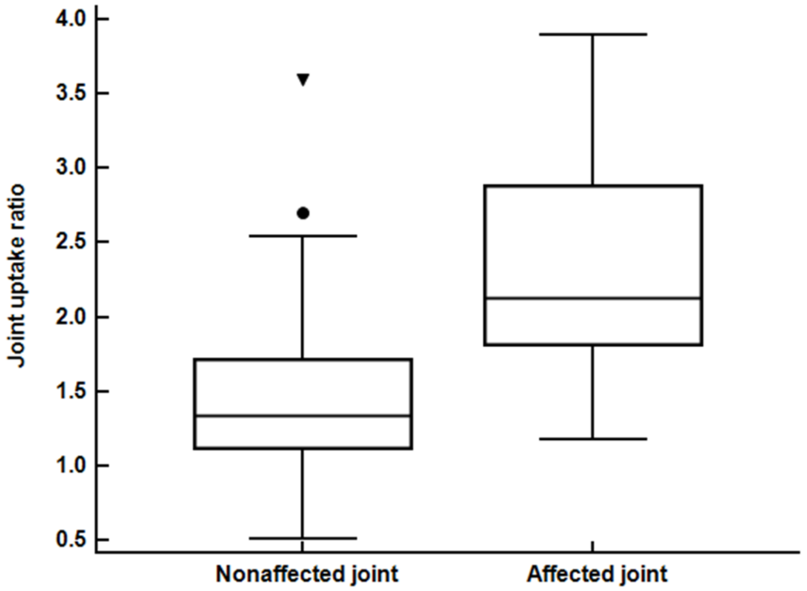

(H) MTP

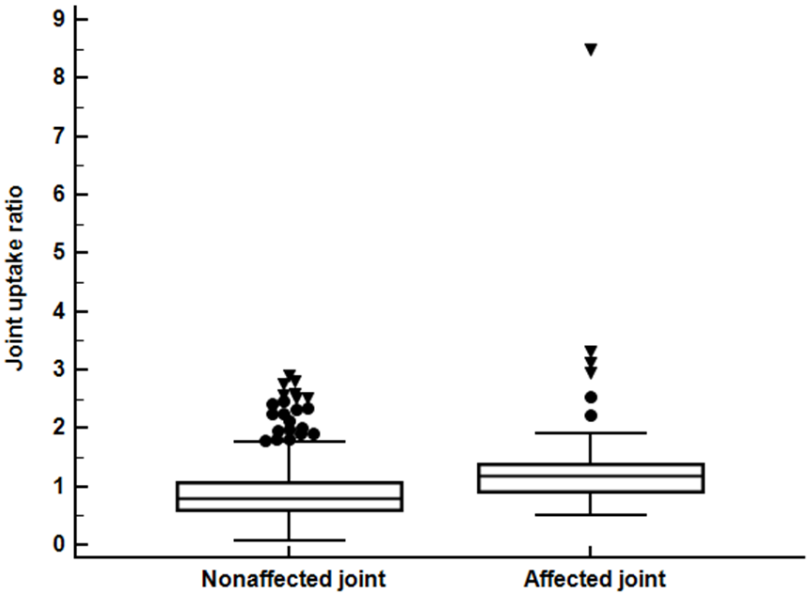

(I) Toe IP

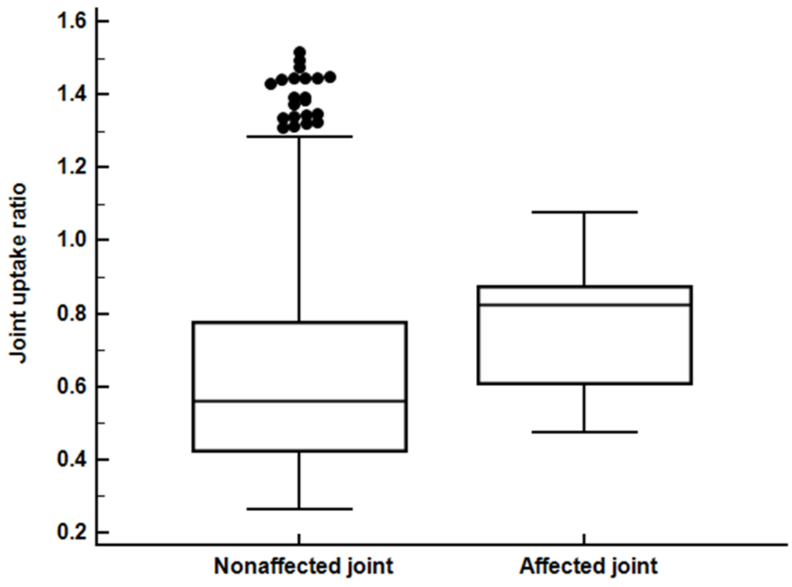

(J) Wrist

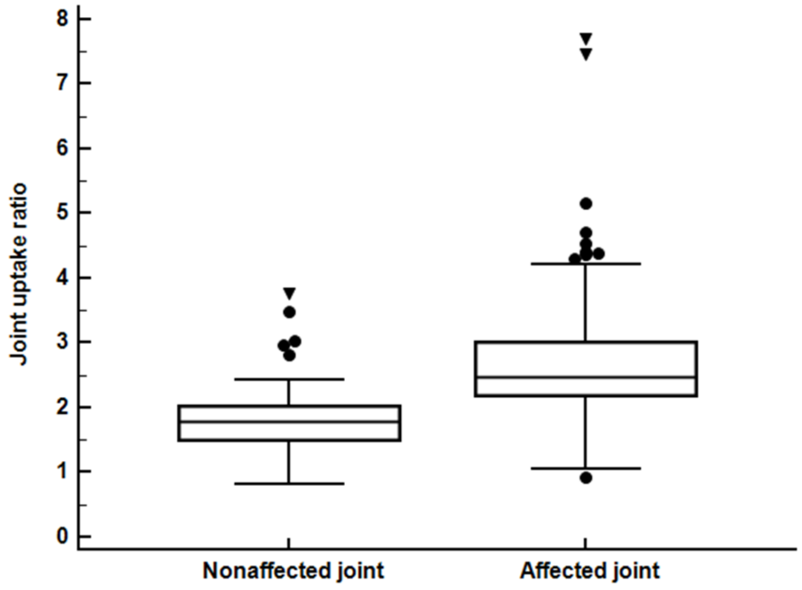

(K) Thumb IP

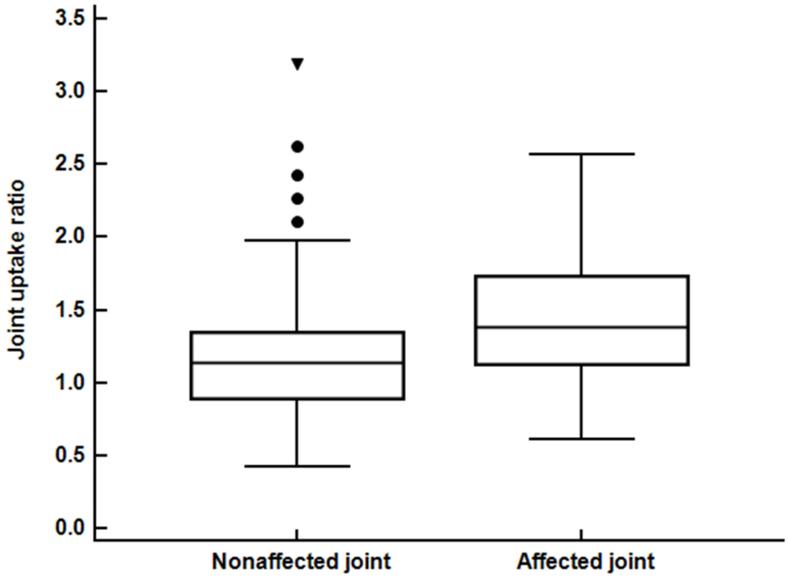

(L) MCP

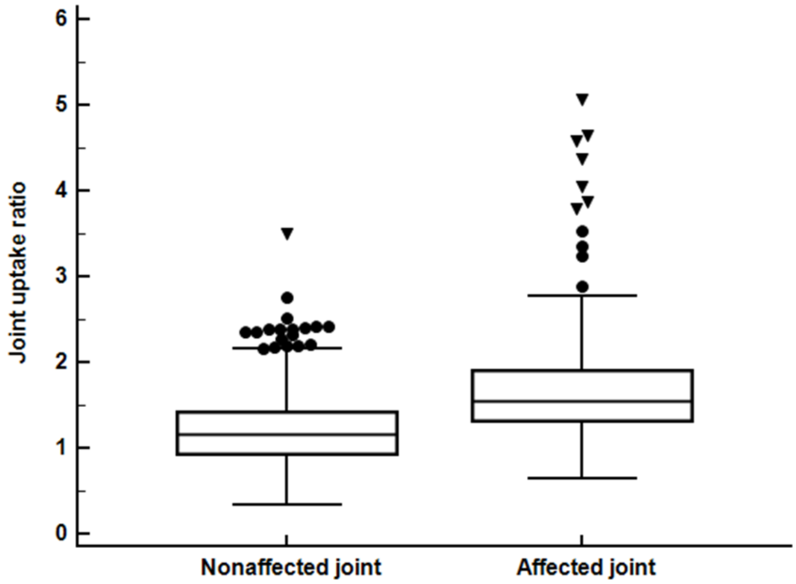

(M) Finger PIP

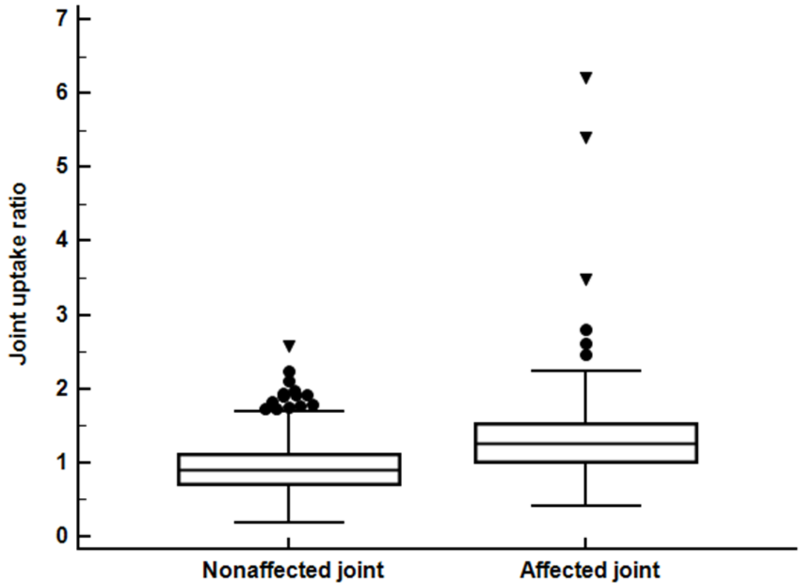

(N) Finger DIP

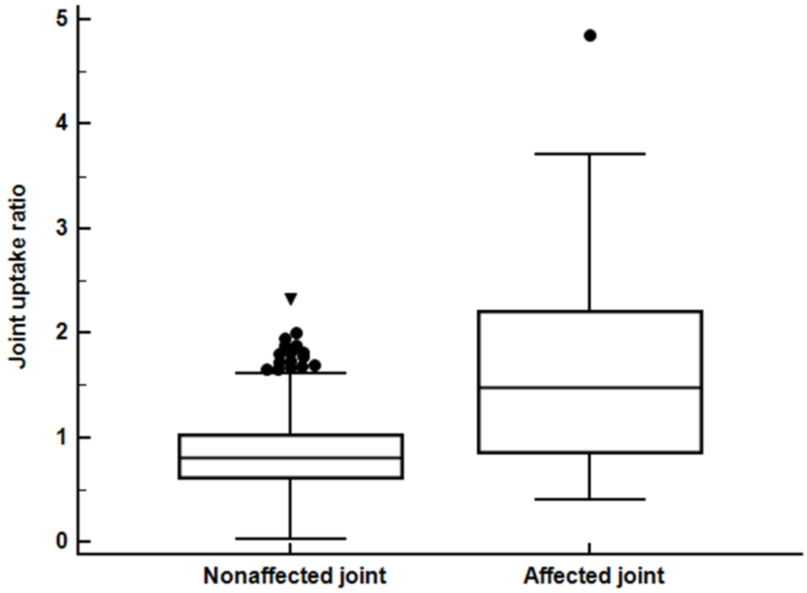

Supplementary Figure. S2. Comparison of receiver operating characteristic curves for the visual analysis of two readers (Reviewer 1 and Reviewer 2) and the joint uptake ratio in detecting affected joints on should er (**A**), sternoclavicular (**B**), elbow (**C**), knee (**D**), sacroiliac (**E**), ankle (**F**), tarsal (**G**), metatarso-phalangeal (MTP) (**H**), toe interphalangeal (IP) (**I**), wrist (**J**), thumb IP (**K**), metacarpo-phalangeal (MCP), (**L**), finger pro ximal interphalangeal (PIP) (**M**), and finger distal interphalangeal (DIP) (**N**) joints.

(A) Shoulder

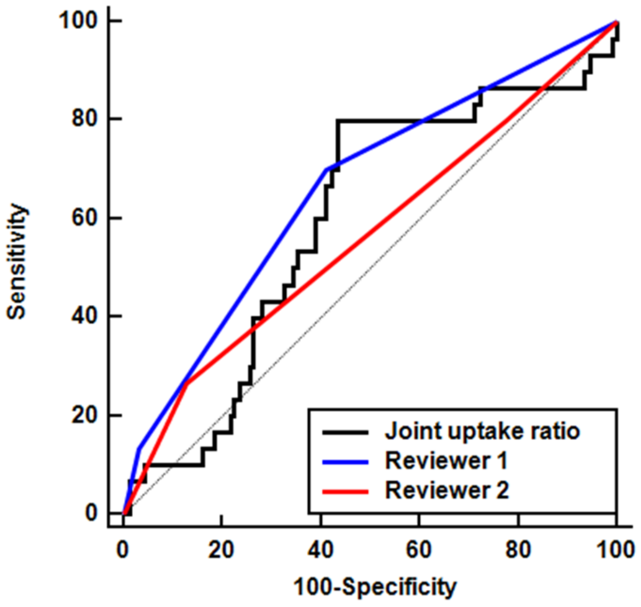

(B) Sternoclavicular

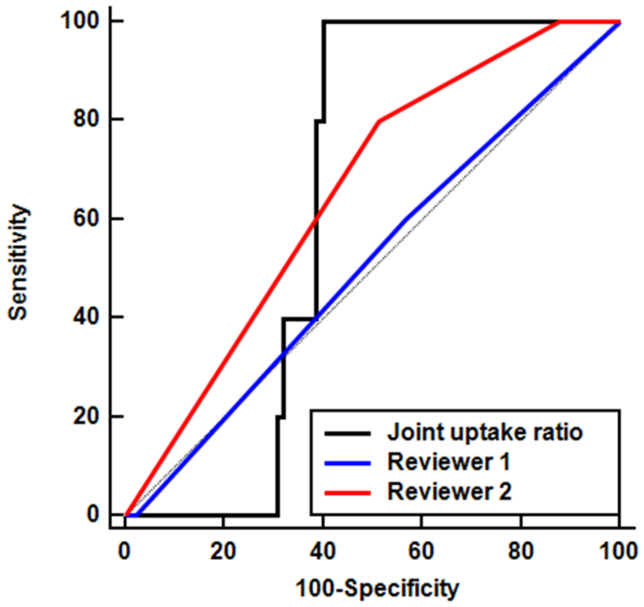

(C) Elbow

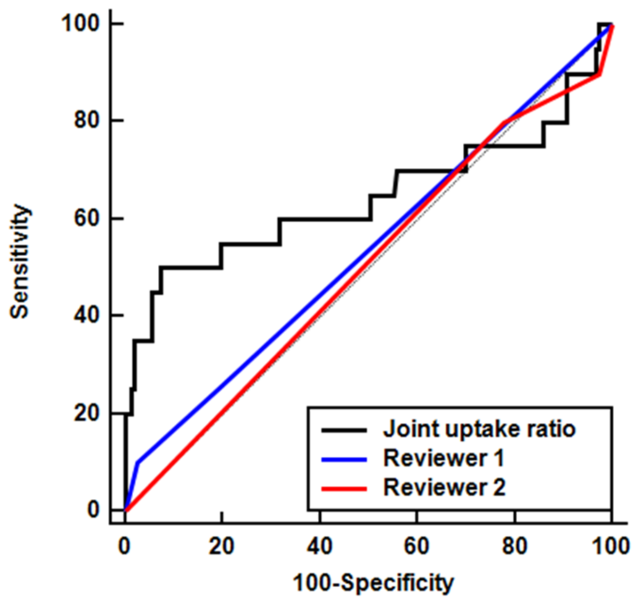

(D) Knee

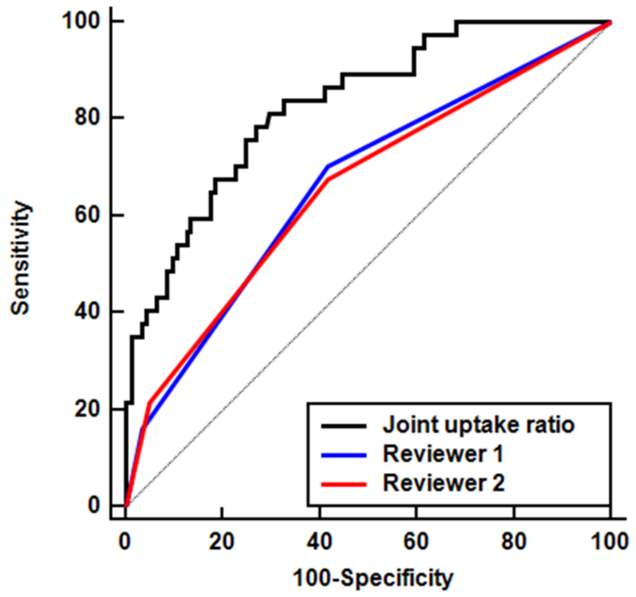

(E) Sacroiliac

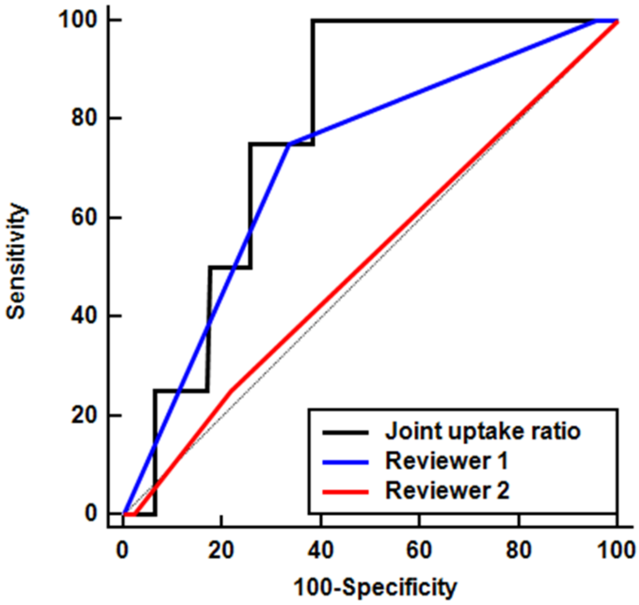

(F) Ankle

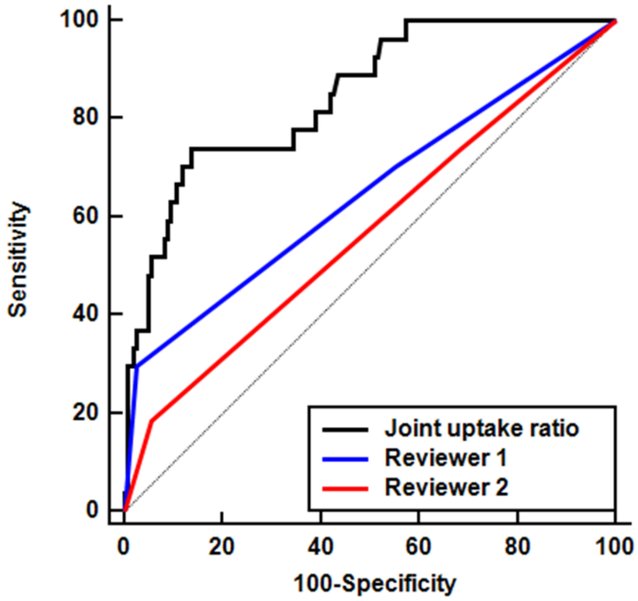

(G) Tarsal

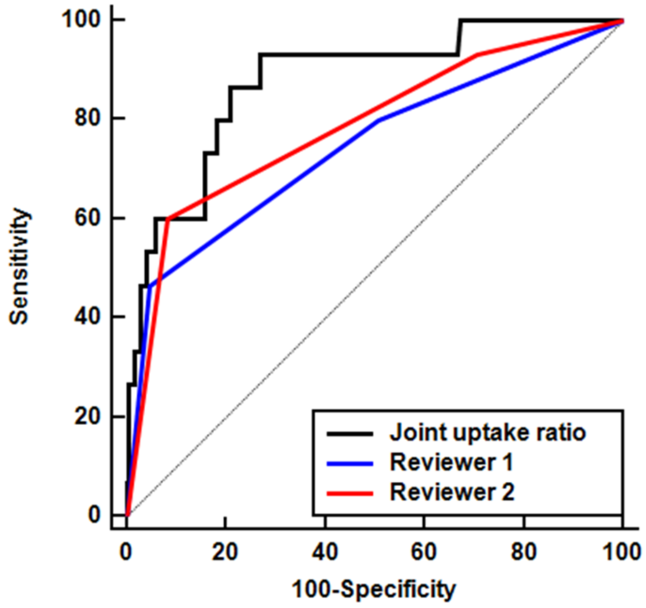

(H) MTP

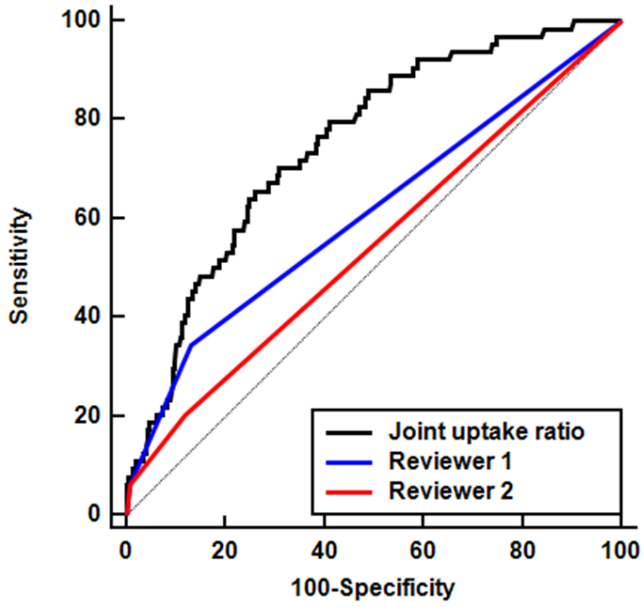

(I) Toe IP

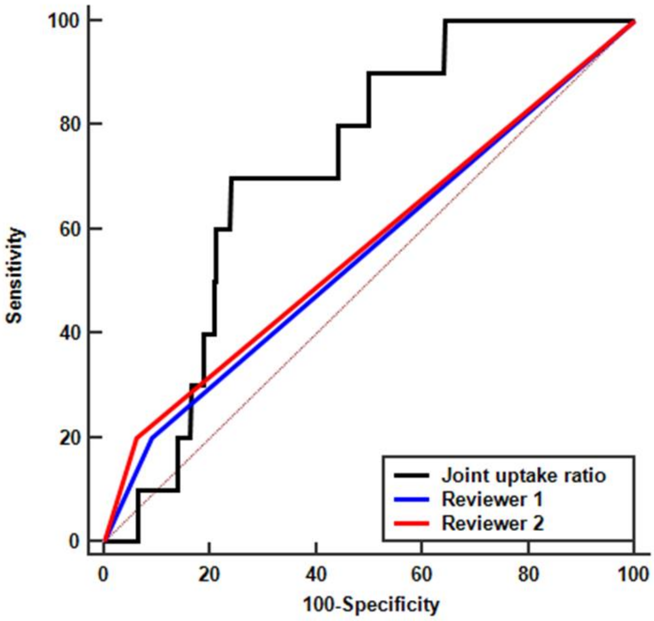

(J) Wrist

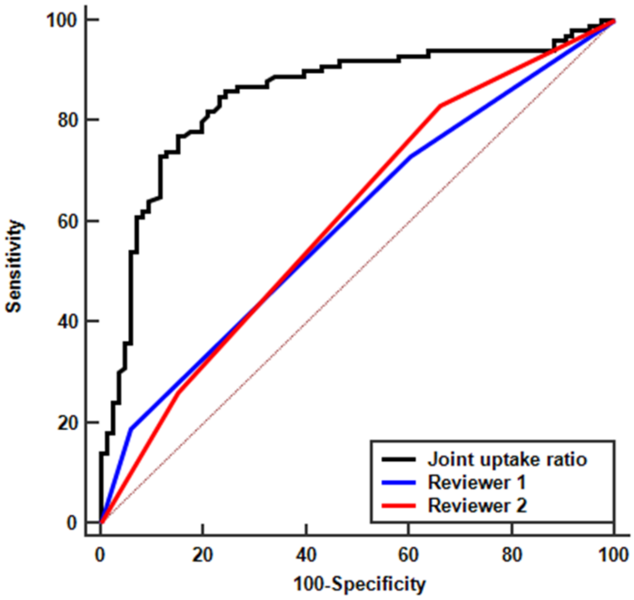

(K) Thumb IP

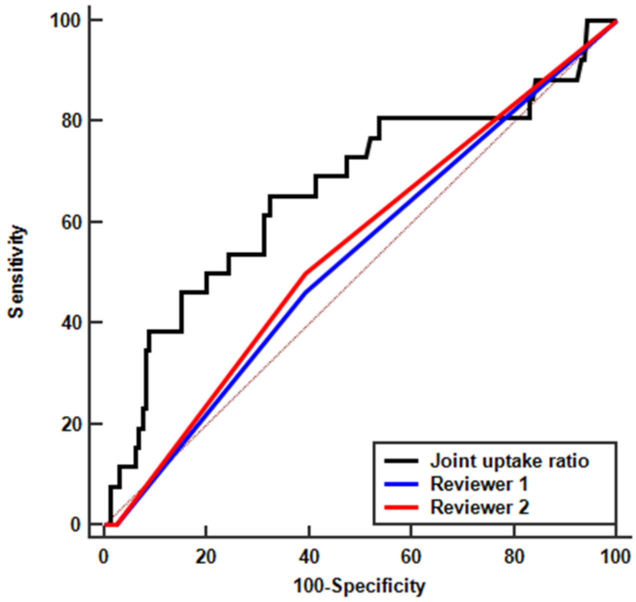

(L) MCP

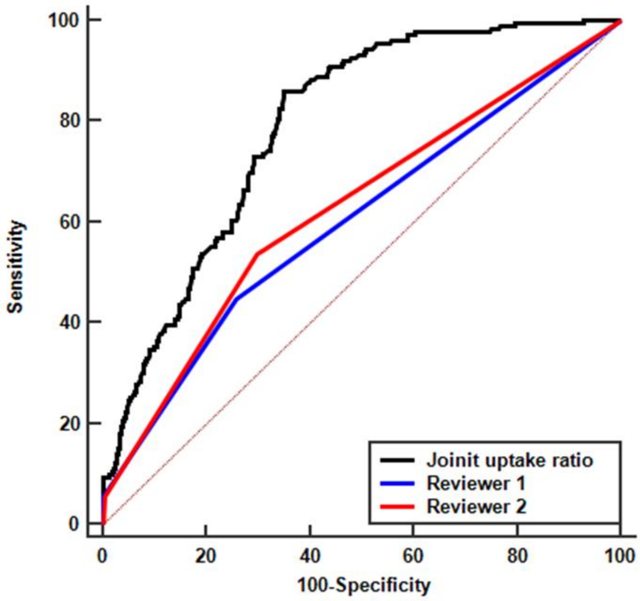

(M) Finger PIP

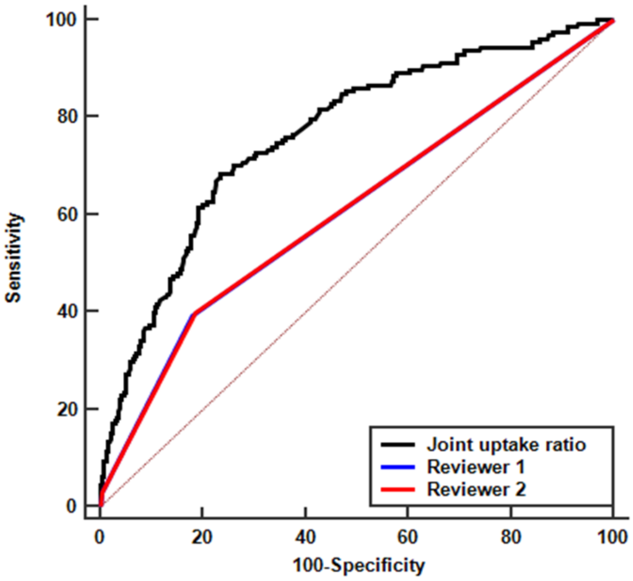

(N) Finger DIP

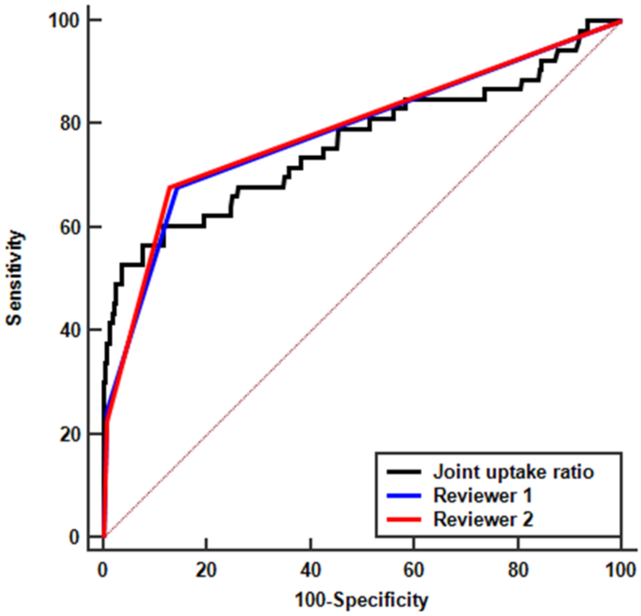

Supplement: Supplementary file 1 [file diagnostics-10-01000-s001.pdf]
